# Development and Validation of a Selective Method to Quantify Low-Molecular-Mass Flavan-3-ols in Grapes and Wines

**DOI:** 10.3390/foods14244257

**Published:** 2025-12-10

**Authors:** Guzmán Favre, Gustavo González-Neves, Diego Piccardo, Yamila Celio-Ackermann, Florencia Pereyra-Farina, Alejandro Cammarota

**Affiliations:** Facultad de Agronomía, Universidad de la República, Avda. Garzón 780. C.P., 12900 Montevideo, Uruguay

**Keywords:** dimethylaminocinnamaldehyde, methylcellulose tannin precipitation assay, proanthocyanidins, condensed tannins, grape and wine polyphenols

## Abstract

Quantifying low-molecular-mass (LMM) flavanols in wines is relevant because these compounds, though typically minor, reflect flavanol structural composition (seed vs. skin contributions) and relate to cultivar and winemaking technique. Their determination is challenging because oligomeric and polymeric tannins interfere with standard spectrophotometric assays. This study introduces a coupled procedure that isolates and selectively quantifies LMM flavan-3-ols by combining the well-established methylcellulose precipitation assay (MCP) to remove oligomers and polymers with dimethylaminocinnamaldehyde (DMAC) determination of the MCP supernatant. The sequential workflow uses DMAC specificity and sensitivity and minimizes interference caused by higher-mass flavanols. Additionally, samples are quantified following dilution in the highly stable MCP supernatant medium. A Small Flavanol Index (SFI, %) is also introduced, expressing the LMM fraction relative to methylcellulose-precipitable tannins and providing a descriptor of flavanol composition. The method was validated in terms of linearity, limits of detection and quantification (LOQ in the supernatant, 1.58 mg L^−1^), precision, and recovery. Applicability is demonstrated in Marselan and Tannat (*Vitis vinifera)*, resolving compositional differences by cultivar, grape tissue (skins vs. seeds), and maceration technique. Compatible with microplate formats and simple instrumentation, this robust analysis enables tandem determination of LMM flavanols and condensed tannins and represents an analytically valuable tool for commercial wineries and research.

## 1. Introduction

Flavanols, including flavan-3-ol monomers such as (+)-catechin and (−)-epicatechin and their polymeric forms (tannins), are key polyphenolic compounds that strongly influence wine quality attributes such as color stability, bitterness, and astringency [1,2,3]. Their concentration and degree of polymerization (DP) critically determine sensory properties and are essential parameters in enological research and wine quality management [3]. Consequently, accurate quantification of these compounds in complex matrices such as grapes and wines is relevant for matrix characterization and for understanding the mechanisms driving phenolic extraction and evolution during winemaking [4]. Analytically, this task remains challenging due to the high chemical heterogeneity and the broad degree-of-polymerization distribution of tannins [5,6]. Although chromatographic methods (HPLC coupled to adequate detectors) provide precise quantification of monomeric and low-oligomeric flavan-3-ols [7,8,9], they are time-consuming, require expensive instrumentation, and are not ideally suited to high-throughput screening or routine winery applications. Moreover, chromatographic protocols often involve laborious sample cleanup or chemical depolymerization steps that may underestimate high-DP polymers or complex tannins [10].

As a rapid and affordable alternative, spectrophotometric assays are widely employed. Among them, the 4-dimethylaminocinnamaldehyde (DMAC) assay is highly selective toward the A-ring of flavan-3-ols [11,12,13], providing strong color development for monomers and low oligomers. However, its response is governed by differences in chemical reactivity rather than absolute concentration. That is, the molar absorptivity of the DMAC-flavanol adduct decreases with increasing DP [14]. Consequently, although DMAC yields measurable absorbance in complex matrices, it does not provide a quantitative estimate of either total flavanols or the low-molecular-mass (LMM) fraction. Therefore, in such systems, the signal mainly reflects relative reactivity rather than true concentration.

To specifically quantify the polymeric fraction responsible for most astringency, precipitation-based methods using polymers such as methylcellulose [15,16,17] or proteins such as bovine serum albumin (BSA) [18] were developed and are widely adopted by researchers and practitioners. These assays remain reference standards for assessing precipitable tannins, effectively isolating the large polymeric tannins that drive tactile astringency in wine [5,19]. Thus, neither MCP nor BSA precipitates LMM flavan-3-ols, which remain in the supernatant due to their small size and limited binding affinity. Of the two, MCP provides a more extensive cleanup of condensed tannins and polymeric pigments, whereas BSA leaves small polymeric pigments in solution [5,15,16,18,20].

Although LMM flavanols are usually minor components in wines, this fraction has been associated with bitterness [21], and, through its chemistry, can significantly affect the sensory profile [21,22]. Critically, its levels vary among grape cultivars and between skins and seeds [23,24,25], and depend strongly on winemaking practices such as maceration time [1,2], as well as cultivar-driven characteristics that determine the relative extraction of seed versus skin tannins during vinification [23,26,27]. Indeed, the proportion of skin-derived tannins relative to total tannins has been identified as a relevant chemical factor, correlating with the market price of red wines [26,28]. Therefore, despite being minor, the LMM fraction may serve as a valuable indicator of both cultivar and winemaking technique.

Thus, a methodological gap remains for simple, affordable, and reproducible quantification of the LMM flavanol fraction in grapes and wines. Here this gap is addressed by validating a complementary two-step workflow that combines the well-referred MCP assay to remove condensed tannins [15,16,29] with subsequent DMAC determination [11] in the MCP supernatant, the DMAC_SOB assay, to obtain a quantitative measure of the LMM fraction. In addition to the efficient removal of condensed tannins, the MCP supernatant (SOB) provides a more homogeneous reaction medium for DMAC, effectively minimizing composition-driven variation in response to the relative original wine or extract. Moreover, we propose the Small Flavanol Index (SFI), defined as (DMAC/MCP) × 100, to express the proportion of LMM flavanols relative to the MC reactive tannin content. Applicability is demonstrated in grape extracts and in red wines from two cultivars (Tannat, Marselan, *Vitis vinifera*) produced using extended maceration and pectolytic enzyme addition, two techniques that, respectively, favor seed and skin-derived flavanol extraction [2,30,31], thereby providing a suitable system in which to evaluate the method. Together, the DMAC_SOB assay and the SFI provide a practical, reproducible assessment of grape and wine flavanol composition, with potential extension to other tannin-containing plant matrices.

## 2. Materials and Methods

### 2.1. Overview

Grape and wine samples were analyzed by an MCP-DMAC workflow in which condensed tannins (HMM) were first removed by the methylcellulose precipitation assay (MCP) (adapted from [17]), and the remaining LMM flavanols in the supernatant (SOB) were quantified by the DMAC assay (adapted from [11]). The Small Flavanol Index (SFI) was calculated as (DMAC/MCP) × 100 (expressing the proportion of DMAC-reactive LMM flavanols per unit of MCP-precipitable tannins).

### 2.2. Chemicals and Reagents

All solvents were HPLC grade and all chemicals were analytical grade. Ultrapure water (18.2 MΩ·cm) was used. Commercial (−)-epicatechin (Sigma-Aldrich, St. Louis, MO, USA; E4018; ≥98% purity) was used for calibration and spiking experiments. The DMAC reagent was prepared using 4-dimethylaminocinnamaldehyde (Sigma-Aldrich, St. Louis, MO, USA, >97.5% purity). Methanol (HPLC grade, ≥ 99.9%, Sigma-Aldrich, St. Louis, MO, USA), hydrochloric acid (~37% *w*/*w*, 12 M, density 1.19 g mL^−1^), formic acid (99% purity; Merck, Darmstadt, Germany), methylcellulose (Sigma-Aldrich; 1500 cP), and ammonium sulfate (analytical grade, Carlo Erba Reagents, Val-de-Reuil, France) were used.

### 2.3. Samples and Winemaking

Experiments were conducted in 2025 at the Enology Laboratory (Facultad de Agronomía, Udelar). Marselan and Tannat grapes (*Vitis vinifera*, 2023 vintage) were harvested from a commercial vineyard in Canelones, south of Uruguay. Seed and skin samples were obtained and extracted using an acidified hydromethanolic solution, as detailed by Favre et al. [23]. These extracts were stored at −18 °C until analysis. Wines were elaborated at experimental scale according to the protocol described by Favre et al. [23]. Two maceration techniques were used; 7-day maceration with the addition of a commercial pectolytic enzyme preparation (pectinase activity; Trenolin^®^ Xtract, ERBSLÖH Geisenheim, Germany) added at vatting (0.3 mL per experimental unit; 9 kg of must), and extended maceration for 15 days without enzyme addition. Bottled wines (375 mL green glass) were corked and stored horizontally at 15.0 ± 0.5 °C in darkness until analysis. Wines were 24 months old at the time of analysis.

### 2.4. General Wine Characterization

Wines were analytically characterized prior to the experiments. Mean values of pH, titratable acidity, volatile acidity, ethanol, and reducing sugars are reported in Table 1. Measurements were performed with a Foss™ Infrared Analyzer (Hillerød, Denmark) and Foss Integrator software v4.8.

### 2.5. Methylcellulose Precipitation Assay

Condensed tannins were determined according to the high-throughput methylcellulose precipitation assay [17], with minor adaptations to allow the use of the available multichannel pipette (minimum dispensing volume of 50 μL). Then, 50 μL of sample were used instead of the original 25 μL proposed. Preliminary internal testing confirmed that this substitution did not introduce significant bias within the flavanol concentration range of the samples used. Thus, for each set of samples, two deep-well plates were prepared; a precipitation plate with MC and a control plate without MC. Aliquots of wine or extract (50 µL) were dispensed into wells. The precipitation plate received 500 µL of 0.04% MC solution per well; both plates were then supplemented with 300 µL of saturated ammonium sulfate and 300 µL of ultrapure water. Samples were homogenized between additions, allowed to stand for 10 min, and centrifuged at 2000× *g* rpm for 6 min (Thermo Fisher, Scientific, Zweigniederlassung, Waltham, MA, USA). Supernatants (200 µL) were transferred to UV-transparent microplates and absorbance was read at 280 nm. Tannin concentration (mg L^−1^ epicatechin equivalents) was calculated as the absorbance difference (MC minus control), multiplied by the dilution factor and converted using the calibration curve. Under these conditions, the operational dilution factor for the MCP supernatant (SOB) relative to the original sample was DF = 23, which was used to refer SOB concentrations back to the original matrix when required.

### 2.6. DMAC Assay

The DMAC working solution consisted of 0.1% (*w*/*v*) DMAC in methanol acidified to 1.2 M HCl. The paired well reference (control) solution was methanol acidified to the same final HCl concentration (1.2 M), to match the reaction medium and ensure stability of the ammonium sulfate present in the SOB.

The DMAC assay followed Vivas et al. [11], adapted to 96-well plates. For direct analysis of wines and extracts, samples were diluted 20-fold in HPLC grade methanol. For SOB analysis, no further dilution was applied beyond that inherent to the MCP assay (DF = 23). In each paired measurement, 50 µL of diluted sample were mixed with 250 µL of either DMAC reagent or the acidified methanol control in clear polystyrene plates. The plate reader was set at 25 °C (15 min equilibration). Plates were shaken for 10 s upon insertion and again just before reading. Absorbance at 640 nm was recorded after 10 min. Net DMAC absorbance was calculated as Abs_DMAC–Abs_control. Concentrations were expressed as mg L^−1^ epicatechin equivalents using the appropriate calibration curve and corrected by the overall dilution factor.

#### Direct DMAC vs. DMAC_SOB

For the paired comparison described in Section 3.2.2, we used Marselan wines elaborated by extended maceration, and its respective seed and skin extracts (*n* = 3). Each matrix was measured before MCP (direct DMAC, DF = 20) and after MCP (DMAC on the SOB, DF = 23). The %SOB was computed as the ratio of the measurement after the MCP step to the measurement before the MCP step, expressed as a percentage.

### 2.7. Flavan-3-ol Analysis by HPLC

The HPLC separation, identification, and quantification of flavanols were performed on a HPLC UltiMate™ 3000 (Thermo, Germering, Germany), equipped with DAD (UltiMate^®^ DAD-3000 Detector, Germering, Germany), a Quaternary pump (UltiMate^®^ LPG-3400SD), an Autosampler (UltiMate^®^ WPS-3000(T) SD), and a thermostatted column compartment (UltiMate^®^ TCC-3000SD), and coupled to a Thermo Scientific Dionex Chromeleon 7 Chromatography Data System Version 7.3.1.

The samples were injected (30 µL) on a reversed-phase column Ascentis Express (4.6 mm × 150 mm; 2.7 µm particle; Agilent, Santa Clara, CA, USA) coupled to a pre-column of identical composition (4.6 mm × 5 mm; 2.7 µm particle), thermostatized at 16 °C following a previously described method [32]. Briefly, the chromatographic conditions were as follows: the solvents were solvent A (water/formic acid, 99.9:0.1, *v*/*v*) and solvent B (methanol/formic acid, 99.9:0.1, *v*/*v*). The flow rate was 0.3 mL/min. The linear solvents gradient was as follows: zero min, 93% A and 7% B; 3 min, 93% A and 7% B; 25 min, 68% A, 32% B; 40 min, 43% A, 57% B; 50 min, 33% A, 67% B; 55 min, 3% A, 97% B; 65 min, 3% A, 97% B; 70 min, 93% A and 7% B (with a post-run of 8 min).

The DAD spectra were recorded (200–800 nm) and chromatograms were extracted at 280 nm for quantification. Flavan-3-ols were identified by matching retention times and UV–Vis spectra to authentic (+)-catechin and (−)-epicatechin standards analyzed under the same conditions. These chromatographic conditions match those previously implemented with DAD-QTOF-MS at the Instituto Regional de Investigación Científica Aplicada (IRICA, University of Castilla–La Mancha), as described by Pérez-Navarro et al. [32], supporting consistent peak assignment across platforms. Quantification was performed by external calibration (Section 2.8), and results are reported as epicatechin equivalents.

### 2.8. Calibration, Linearity, and Sensitivity Parameters

Epicatechin stock solutions were prepared by dissolving the analyte in 1 mL of HPLC-grade methanol and bringing to 10 mL with ultrapure water. Working standards were prepared freshly for each method: 10–250 mg L^−1^ for MCP, 0–25 mg L^−1^ for direct DMAC, 0–6 mg L^−1^ for DMAC in SOB, and 10–100 mg L^−1^ for HPLC. All calibration points were run in triplicate. Ordinary least squares (OLS) was used to fit the calibration curves, while R^2^ (coefficient of determination) and residual analysis were used to assess linearity. Limits of detection and quantification were estimated from the calibration slope and the residual standard deviation as LOD = 3.3 σ/slope and LOQ = 10 σ/slope, in line with international guidelines. Back-calculated concentrations were used to summarize %RSD and absolute bias across the decision range. The full regression equations, back-calculation tables, and calibration plots are presented as follows: MCP—Figure S1 and Table S1 (Supplementary Information); Direct DMAC—Figure S2 and Table S2 (Supplementary Information); HPLC—Figure S3 and Table S3 (Supplementary Information). Results for DMAC in the SOB are presented in Section 3 (Results and Discussion). (−)-Epicatechin was used as the calibrant because it is a major constituent of the flavan-3-ol pool in grapes and wines and aligns units with the MCP assay. Quantities are expressed as epicatechin equivalents as a practical single-standard convention, similar to the use of malvidin-3-glucoside equivalents for anthocyanins, recognizing that response factors can vary among individual flavan-3-ols [33].

### 2.9. Recovery Studies

Method accuracy and matrix cleanup efficiency were evaluated by spiking an extended macerated Marselan wine and the corresponding seed and skin extracts (see Section 2.3) with epicatechin prior to the MCP step. Fortification levels were 3, 15, 30, 51 and 75 mg L^−1^. Spikes were prepared by mixing 0.3 mL of the appropriate epicatechin working solution with 0.7 mL of the corresponding matrix to a final volume of 1.0 mL. For each matrix, an unspiked sample and a dilution control (spiked with the same volume of solvent, without epicatechin) were included. Fortified samples underwent the full MCP procedure and the SOB was quantified by DMAC. All measurements were performed in triplicate. In parallel, cross-validation was conducted by HPLC, as explained above (Section 2.7). For each matrix, C_recovered (mg L^−1^) was regressed against C_added (mg L^−1^) by OLS, and 95% confidence intervals for slope and intercept were computed from residual variance.

Intermediate precision was assessed by running the 0–6 mg L^−1^ DMAC–SOB calibration again on a separate day, and measuring spikes at 7.5 and 30 mg L^−1^ across the three matrices. Inter-day slope and intercept agreement, LOD/LOQ, and recoveries are summarized in Tables S4 and S5.

### 2.10. Data Analysis and Statistics

All calculations and plots were performed in R [34] within RStudio [35], using the packages tidyverse [36] for data handling and plotting, emmeans [37] for post hoc comparisons (Tukey HSD at 5%), and multcomp [38] for multiple-comparison procedures. Group comparisons for tables and figures were evaluated by ANOVA, followed by Tukey HSD at 5%.

## 3. Results and Discussion

### 3.1. Suitability of the Methodology for Flavan-3-ol Analysis in MCP-Supernatant

**Figure 1:**
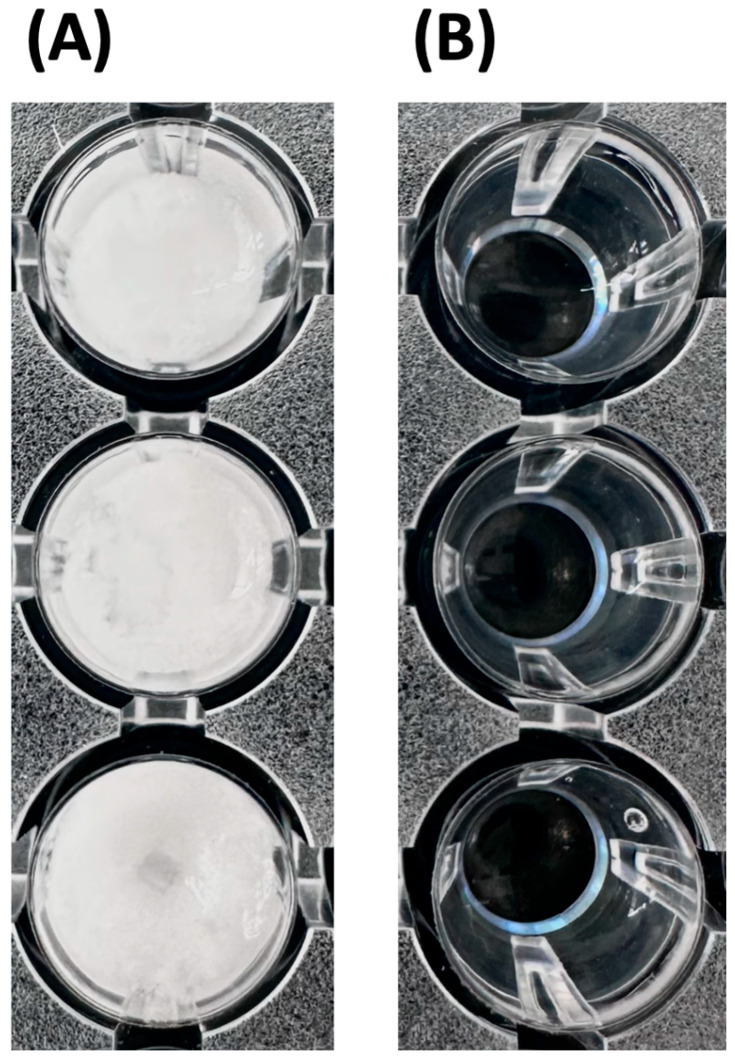
Effect of methanol acidification on the DMAC assay control wells. Visual demonstration of the necessity of an acidic solvent for accurate analysis in the DMAC assay applied to the MCP supernatant. (**A**) Control wells containing non-acidified methanol as the solvent, resulting in ammonium sulfate precipitation. (**B**) Control wells where the methanol solvent was acidified (1.2 M HCl), enabling ammonium sulfate solubilization and ensuring correct measurements.

The MCP-DMAC workflow was first evaluated for chemical compatibility and performance in quantifying LMM flavanols in the methylcellulose supernatant (SOB) from wines and grape extracts. In all cases, LMM components in SOB reacted with DMAC to yield measurable color that was quantified spectrophotometrically. A critical operational modification was required for the paired-well control used to correct non-specific absorbance at 640 nm. The methanolic control solvent must be acidified to match the reaction medium. Without acidification, immediate precipitation of ammonium sulfate occurs, which prevents accurate spectrophotometric readings. Accordingly, both the DMAC reagent and the control solvent were acidified, ensuring stable, reliable paired-well correction (see Materials and Methods Section 2.6; Figure 1).

### 3.2. Calibration Curves for Flavanol Quantification

All methods showed excellent linearity within their working ranges (see calibration details in Materials and Methods and the corresponding Figures and Tables). Given the study focus on the flavanol LMM fraction, subsequent analyses refer to the DMAC_SOB calibration. Full regression parameters for the remaining methods are provided in the Supplementary Information.

#### 3.2.1. Performance of the DMAC_SOB Calibration Curve

**Figure 2:**
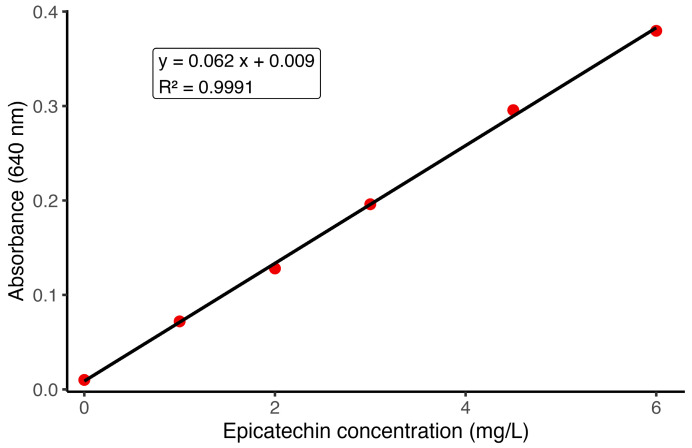
Calibration curve for the MCP supernatant DMAC analysis. Calibration curve prepared using epicatechin standards across the 0.0–6.0 mg L^−1^ concentration range. Each standard was measured in triplicate. The strong linearity of the curve confirms method suitability.

**Figure 3:**
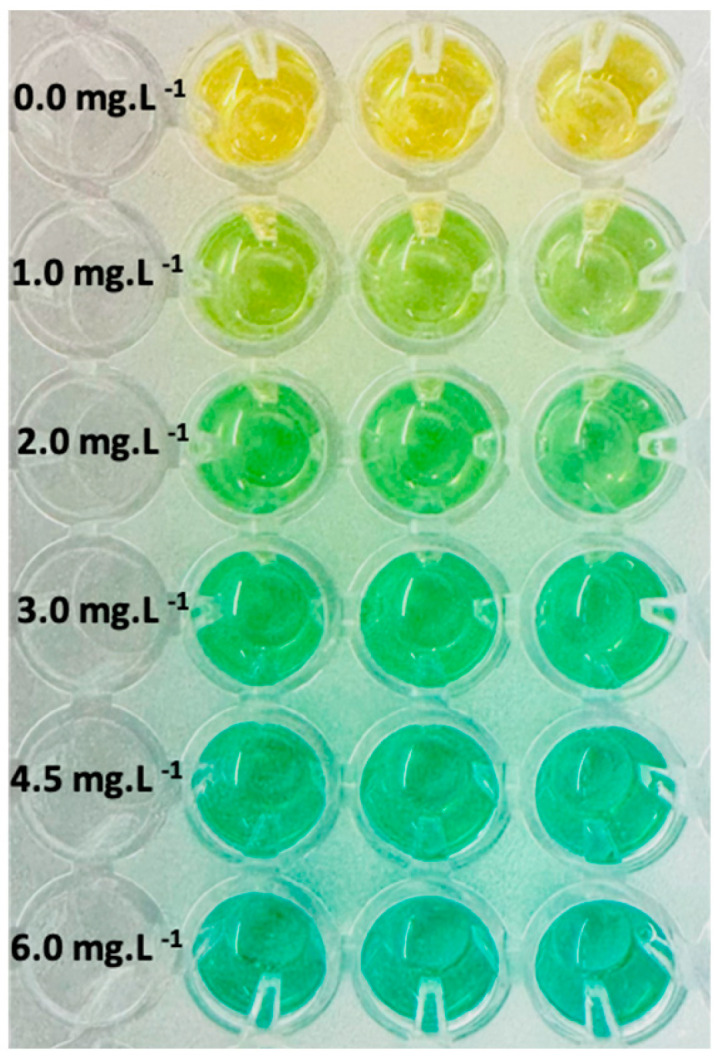
Color development of the DMAC-flavanol reaction as a function of (−)-epicatechin concentration in a 96-well microplate. Triplicate rows correspond to each standard concentration used to construct the calibration curve. Higher (−)-epicatechin levels produce progressively stronger green coloration.

The calibration in MCP supernatant showed excellent proportionality across the working range (Figure 2 and Figure 3, Table 2). Slope and residual dispersion yielded LOD = 0.52 mg L^−1^ and LOQ = 1.58 mg L^−1^ (Table 3), supporting quantitative analysis from approximately 2 to 6 mg L^−1^ in SOB. Referred to the original sample, applying DF = 23 corresponds to roughly 46–138 mg L^−1^ (LOQ ≈ 36 mg L^−1^). The small positive intercept (0.009 A.U.) indicates a stable background relevant only near the LOQ. Back-calculated concentrations showed %RSD of 2–5% with absolute bias ≤ 4.4% in the 2–6 mg L^−1^ range (Table 2). At 1.0 mg L^−1^, %RSD was approximately 10%, consistent with operation below LOQ and indicating semi-quantitative performance at best. As described in Section 2.9, the calibration performed on a separate day yielded comparable parameters; see Tables S4 and S5.

#### 3.2.2. Direct DMAC vs. DMAC_SOB According to the Matrix Analyzed

**Figure 4:**
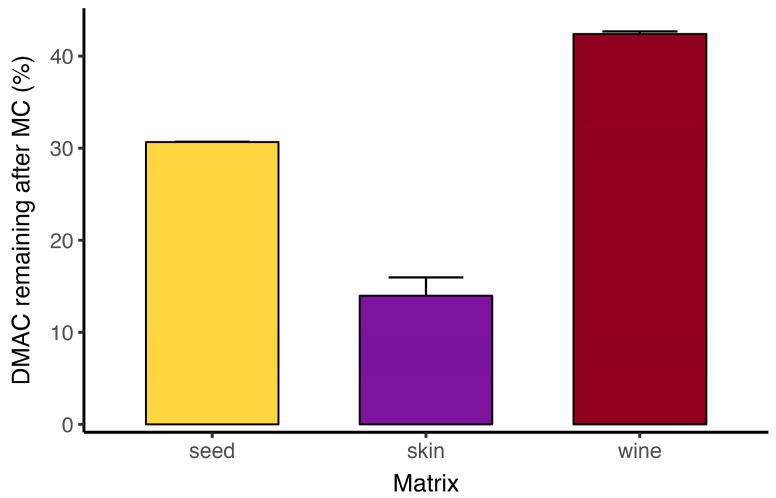
Flavanols reactive to DMAC in the methylcellulose supernatant (SOB) for Marselan wines produced by extended maceration and the corresponding seed and skin extracts (*n* = 3 per matrix), expressed as a percentage of the direct DMAC determination (before methylcellulose precipitation).

Paired measurements obtained before and after the methylcellulose precipitation step (illustrated here for Marselan wines and the corresponding skin and seed extracts) show that applying DMAC to the MCP supernatant (DMAC_SOB) markedly reduces the signal relative to direct DMAC measured on the same sample (Figure 4). Expressed as a percentage of the direct DMAC response, the fraction remaining in the supernatant averaged ~31% for seed extracts, ~14% for skin extracts, and ~42% for wine. This indicates that a substantial proportion of the direct DMAC signal arises from MCP precipitable material, namely oligomeric and polymeric proanthocyanidins, rather than exclusively from LMM flavan-3-ols.

These results therefore highlight an important limitation of interpreting direct DMAC as a total flavanol measurement in complex matrices that contain mixtures of monomers, oligomers, and polymers. The DMAC reagent reacts with flavan-3-ol units across this range, but the response is known to be strongly dependent on proanthocyanidin structure and degree of polymerization [13]. As a result, the direct DMAC signal reflects not only concentration but also structural features of the sample, and consequently varies with matrix type. Thus, the results observed in Figure 4 are consistent with the expected distribution and structural characteristics of flavanols across these matrices [23,25]. Overall, the data demonstrate the value of the MCP cleanup step to isolate the supernatant pool and improve the quantitative interpretability of DMAC for comparative flavanol assessment in grape and wine samples.

### 3.3. Recovery Experiments: Evaluation of Accuracy and Matrix Effects

#### 3.3.1. Analytical Accuracy and Bias

Spike recoveries in the MCP supernatant (SOB) were evaluated in skins, seeds, and wine to assess accuracy and matrix effects. Skins and seeds showed quantitative recovery without proportional or constant bias, clustering near 100% across the working range. Wines displayed a positive intercept (fixed offset) that was most evident at low levels and negligible by 50–75 mg L^−1^ (Table 4 and Table S6). Consistent with this, mid-to-high spikes (30–75 mg L^−1^) were generally excellent in all matrices, whereas wines showed mild over recovery at intermediate levels (15–30 mg L^−1^). Nonetheless, absolute amounts remained representative of the added (−)-epicatechin.

Importantly, near the LOQ (LOQ = 1.58 mg L^−1^ in SOB), even a small matrix-derived fixed offset can produce a disproportionately large positive bias, and recovery calculations (native subtraction) inflate relative variance. This explains the largest SD at the 3 mg L^−1^ spikes.

Despite the overall adequate robustness of the method, the tendency towards systematic overestimation, particularly observed in the wine matrix, warrants further discussion. The influence diminishes rapidly with concentration, and precision and bias improve markedly at mid-to-high levels. The positive bias observed in the recovery calculations is likely rooted in an interaction effect between the added (−)-epicatechin standard and the complex components of the wine matrix. This phenomenon should be considered, particularly when working with low LMM flavanol contents, and merits further investigation. Consequently, when low LMM flavanol concentrations are anticipated, increasing the initial volume of wine used in the MCP precipitation step represents a potential strategy to ensure that SOB concentration falls within the range where precision and bias are demonstrably optimal. Importantly, methylcellulose precipitation does not approach capacity except at very high tannin concentrations [15]. Thus, increasing the sample volume in the MCP assay should not alter precipitation selectivity or introduce bias. Overall, the results confirm efficient isolation of the LMM flavanol fraction by the MCP step and analytical robustness within the practical working range, with a defined limitation at the lowest spike, particularly in wines. For strict quantification near the LOQ, confirmation with a fully quantitative chromatographic method is advisable. For sample characterization or comparative profiling across samples or treatments, the present DMAC_SOB workflow remains fit for purpose, provided this low-level limitation is considered.

#### 3.3.2. Cross-Validation with Reference Method (HPLC)

**Figure 5:**
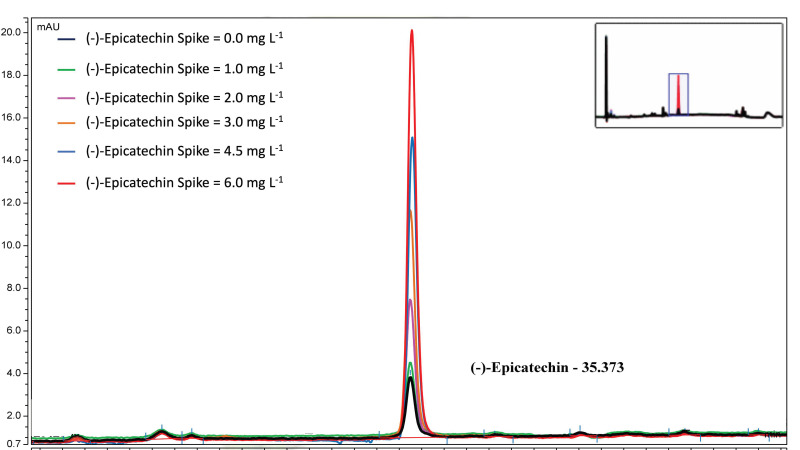
Overlaid HPLC chromatograms (280 nm) of the (−)-epicatechin standard spikes recovered in the methylcellulose assay supernatant of a Marselan seed extract.

Spike-recovery experiments were cross-validated by HPLC (Table 5 and Table S7). Seed and skin presented near ideal agreement (seed: slope = 1.006, R^2^ = 0.9996; skin: 1.002, R^2^ = 0.9997), confirming that MCP precipitation did not remove the spiked epicatechin from the LMM fraction (Table 5, Figure 5). In wines, an over-recovery was observed (slope = 1.56, R^2^ = 0.9997), consistent with a positive chromatographic interference that increases peak area. Although SPE (C18 alone or C18 plus cation exchange) is commonly employed to reduce chromatographic interferences [32], it also removes a small but quantifiable fraction of flavan-3-ol, and was thus unsuitable for the specific objective of our recovery assessment. Because the positive bias observed in wine stems from interference components rather than loss during MCP precipitation, and seed and skin showed quantitative recovery, it is reasonable to infer that MCP does not significantly precipitate the spiked (−)-epicatechin in wines either.

The analytically negative concentration and recovery values observed in the skin matrix at the 3 mg L^−1^ spike level (Table 5) are a result of the extreme measurement variance near the LOQ. At this low concentration, the measured signal of the spiked sample was marginally lower than the native sample, leading to a negative result when applying the recovery formula. This highlights the limitation in precision of the automated method when analyzing highly complex matrices near the detection limit. As the spike level increased, this variance was overcome, and the recovery values rapidly normalized, confirming the suitability of the method for concentrations within the established working range.

### 3.4. Assessing the Ability of the DMAC_SOB Method to Detect Flavanol Differences Across Grape Matrices, Cultivars, and Winemaking Techniques

To demonstrate applicability, LMM flavanols (DMAC_SOB), HMM flavanols (MCP), and the SFI were quantified in hydromethanolic extracts of grape seeds and skins and in the corresponding wines produced using two maceration techniques (Table 6). In the extracts, the matrix was the primary driver. Seeds from Marselan and Tannat showed similar LMM and HMM contents (*p* > 0.05), yielding an SFI of approximately 6%. Skins displayed markedly lower LMM (20.01–28.84 mg L^−1^) but roughly doubled the HMM content relative to seeds (Table 6), producing an index more than fourfold lower and highlighting tissue-specific compositional differences consistent with prior HPLC based reports [23,24]. Tannat skins presented slightly higher DMAC values than Marselan, while HMM levels were comparable between cultivars.

In wines, the effect of the maceration technique depended on cultivar. For Marselan, a cultivar with unusually low extraction from skins [23], extended maceration increased both LMM and the SFI relative to enzyme treatment, consistent with enhanced extraction of seed-derived LMM during prolonged contact. In Tannat, enzyme-assisted maceration yielded higher LMM than extended maceration but particularly increased HMM, consistent with greater extraction of skin material, thus lowering the SFI. When comparing cultivars within the same maceration strategy, Marselan consistently retained higher LMM concentrations and a greater SFI than Tannat (Table 6), suggesting a larger contribution of seed-derived tannins to the final wine, in line with known cultivar characteristics [23].

Building on these applicability results, we next position the workflow relative to existing methods. In comparison with existing approaches, the DMAC_SOB workflow targets the LMM fraction remaining after removal of polymeric tannins, thereby complementing the methylcellulose-precipitable tannin assay [15,16] and enabling a simple structure-related index (the SFI), because both readouts are obtained sequentially and reported in epicatechin equivalents. Protein-precipitation methods such as the Adams–Harbertson bovine serum albumin assay [18] provide a different operational definition based on tannin–protein interactions and are highly informative, but flavanol precipitation is less complete than with methylcellulose, leaving a different pool in solution [16,18,19]. Among phenolic-aldehyde colorimetric assays, vanillin is historically common, but its response depends strongly on flavanol structure; DMAC is chemically related yet offers higher sensitivity and better repeatability for routine low-level detection [33]. Chromatographic approaches (HPLC; depolymerization in the presence of a nucleophilic agent plus HPLC, size exclusion chromatography, etc.) provide richer speciation but are time-and instrument-intensive and are not suited to high-throughput screening [8,9]. We also note the Vivas polymerization index concept [11], which relies on destructive hydrolysis, a process that is highly incomplete and sensitive to matrix effects. To our knowledge, no rapid spectrophotometric method presently isolates and quantifies the LMM fraction in these matrices. The method proposed here (DMAC_SOB) is intended to fill that practical gap by taking advantage of the high removal of condensed tannins by the MCP assay and the high sensitivity of DMAC for flavan-3-ols. Additionally, the sample is quantified following dilution in the highly stable MCP supernatant medium (SOB). This assay remains complementary to chromatographic confirmation when strict quantification is required.

Ongoing research involves evaluating the method across a broad range of cultivars, both to characterize their flavanol profiles and to further quantify the discriminatory power of the method for distinguishing samples.

The application of the method during winemaking will also be considered, specifically to evaluate the extraction of flavanols through maceration in different cultivars and under varying winemaking techniques. Furthermore, evaluating the performance of the method in additional tannin-containing matrices such as tea, cacao, enological tannins, and other botanical extracts will also be interesting. Because DMAC reactivity can vary with tannin class, matrix-specific checks are required before implementation outside grape and wine. In grapes and wines, the dominant flavan-3-ol building blocks (catechin, epicatechin, (epi)gallocatechin and their gallates) assemble into proanthocyanidins. Although linkage patterns and degrees of polymerization vary, these families are well-described [39,40]. For matrices with different tannin compositions, additional benchmarking is warranted.

Practical modifications to the present proposed method could be explored, but the fundamental principles of both involved assays and response linearity must be rigorously preserved. One such modification (as discussed in Section 3.3.1) could include increasing the sample volume in the preceding MCP assay in order to increase LMM flavanol concentrations in the SOB when low contents are expected, thereby aiming to mitigate limitations observed at low concentrations.

## 4. Conclusions

The DMAC_SOB assay provides a practical means to quantify low-molecular-mass (LMM) flavanols while retaining the established measure of precipitable tannins, yielding a more complete description of flavanol composition with widely available laboratory instrumentation. Beyond analytical validation, the approach resolved matrix, cultivar, and maceration effects, and the Small Flavanol Index (SFI) proposed here offered a compact descriptor linking the MCP and DMAC_SOB for comparative studies across samples and vintages. Although the study implemented a microplate, high-throughput format, the measurement can also be performed with a conventional spectrophotometer. The proposed method is robust and fit for purpose for the rapid and informative assessment of flavan-3-ol; however, the observed overestimation and high variance near the limit of quantitation must be considered, particularly when performing strict absolute quantification in complex matrices like wine. Overall, the method is suitable for routine use in research and shows promise for broader application across tannin-containing matrices. Expanding the evaluation to additional cultivars and vinification conditions will broaden applicability.

## Figures and Tables

**Table 1:** General analytical parameters of the red wine samples used for method validation.

Wine	Ethanol (% *v*/*v*)	pH	Titratable Acidity (g/L)	Volatile Acidity (g/L)	Residual Sugars (g/L)
Marselan EZ	13.6 ± 0.2 ^b^	3.63 ± 0.03 ^b^	3.70 ± 0.05 ^b^	0.23 ± 0.02 ^b^	3.86 ± 0.10 ^a^
Marselan ME	13.3 ± 0.2 ^c^	3.73 ± 0.02 ^a^	3.41 ± 0.03 ^c^	0.31 ± 0.03 ^a^	2.30 ± 0.37 ^c^
Tannat EZ	14.6 ± 0.1 ^a^	3.56 ± 0.04 ^c^	3.90 ± 0.09 ^a^	0.27 ± 0.02 ^ab^	3.24 ± 0.34 ^b^
Tannat ME	13.8 ± 0.2 ^b^	3.66 ± 0.05 ^b^	3.68 ± 0.10 ^b^	0.29 ± 0.06 ^ab^	2.54 ± 0.40 ^c^

Notes: Values are expressed as the mean ± standard deviation (*n* = 3). Different letters within the same column indicate statistically significant differences between the wines based on the Tukey honestly significant difference (HSD) test (*p* < 0.05). Titratable acidity and Volatile acidity are expressed in gram of sulfuric acid per liter. EZ denotes wines elaborated with pectolytic enzymes addition at vatting, 7 maceration days; ME denote wines elaborated by extended maceration, 15 maceration days without enzymes addition.

**Table 2:** Evaluation of precision and accuracy by calibration level (back-calculated concentration) for the DMAC reaction in methylcellulose supernatant.

Standard Concentrations	Mean calc. (mg L^−1^)	SD (mg L^−1^)	%RSD	Bias (%)
1.0	1.01	0.105	10.37	1.41
2.0	1.91	0.064	3.35	−4.38
3.0	3.00	0.153	5.10	0.10
4.5	4.60	0.082	1.79	2.26
6.0	5.95	0.307	5.16	−0.85

Notes: Mean calc., mean concentration back-calculated using the calibration curve (*n* = 3 replicates per level). SD, standard deviation. RSD, relative standard deviation. Bias, relative error calculated as: 100 × (Mean calc.−Nominal Concentration)/Nominal Concentration.

**Table 3:** Evaluation of DMAC-SOB calibration curve parameters.

Parameter	Value (SOB)	Original Sample (×23)
Slope (b_1_)	0.062 A.U. (mg L^−1^)^−1^	
Intercept (b_0_)	0.009 A.U	
Residual SD	0.00986 A.U	
R^2^	0.99470 A.U	
LOD	0.52180 mg L^−1^	12.00 mg L^−1^
LOQ	1.58100 mg L^−1^	36.37 mg L^−1^

Notes: Parameters of the DMAC-SOB calibration curve (range 0–6 mg L^−1^), determined by Ordinary Least Squares (OLS) regression. *n* = 6 represents the number of distinct calibration points used for the regression (each point based on three analytical replicates). The Residual SD (sy/x) is expressed in Absorbance Units (A.U.). The LOD and LOQ were calculated using the ICH guideline (3.3⋅sy/x/b1 and 10⋅sy/x/b1, respectively). Original Sample values indicate the limits expressed in terms of the original sample concentration, considering the 23-fold dilution factor (×23) applied during the MCP supernatant preparation.

**Table 4:** Recovery and calculated concentration of (−)-Epicatechin in different matrices (Skin, Seed, and Wine) following the MCP-DMAC analytical procedure.

Matrix	Spike (mg L^−1^)	SOB Recovery (mg L^−1^)	%Rec.
Skin	3	2.6 ± 0.57	86.1 ± 52.2
	15	13.5 ± 0.78	89.8 ± 5.2
	30	30.6 ± 3.65	102.1 ± 12.2
	51	50.5 ± 12	99.1 ± 23.5
	75	74.3 ± 1.83	99.1 ± 2.4
Seed	3	4.1 ± 1.57	135.3 ± 52.2
	15	9.4 ± 6.52	62.7 ± 43.5
	30	30.3 ± 2.61	100.8 ± 8.7
	51	45.7 ± 5.74	89.7 ± 11.3
	75	76.2 ± 2.35	101.6 ± 3.1
Wine	3	8.9 ± 5.74	295.1 ± 191.3
	15	18.8 ± 0.0	125.4 ± 0.0
	30	34.9 ± 1.3	116.2 ± 4.3
	51	52.0 ± 7.3	102 ±14.3
	75	76.7 ± 0	102.3 ± 0.0

Note: Values represent the mean concentration and standard deviation (±SD) from three analytical replicates (*n* = 3 per level). Spike, nominal concentration added (mg L^−1^). %Rec., percentage recovery, calculated as 100 × (C_Recovered_/C_Added_). The matrices studied included Marselan wine and its corresponding hydromethanolic extracts of seed and skin. SOB recovery refers to the recovered concentration of (−)-epicatechin in the MCP supernatant.

**Table 5:** Recovery and calculated concentration of (−)-epicatechin in MCP supernatants by HPLC analysis by spike level and matrix.

Matrix	Spike Level (mg L^−1^)	SOB recovery (mg L^−1^)	% Rec.
seed_sob	3	2.50 ± 0.12	83.00 ± 4.25
	15	17.20 ± 0.82	115.00 ± 5.76
	30	30.09 ± 1.57	101.95 ± 5.48
	51	50.00 ± 2.65	98.00 ± 4.88
	75	75.10 ± 3.75	100.00 ± 4.30
skin_sob	3	−1.60 ± 0.08	−55.00 ± 2.82
	15	5.70 ± 0.32	38.00 ± 1.63
	30	25.01 ± 1.30	85.00 ± 4.35
	51	48.12 ± 2.45	94.00 ± 5.12
	75	80.00 ± 4.01	107.00 ± 5.37
wine_sob	3	6.30 ± 0.35	211.00 ± 10.45
	15	19.50 ± 0.99	130.00 ± 7.10
	30	44.80 ± 2.21	150.00 ± 7.42
	51	72.60 ± 3.79	153.00 ± 7.49
	75	114.80 ± 5.71	153.00 ± 7.85

Notes: Values are reported as the mean ±SD (Standard Deviation) from three analytical replicates (*n* = 3 per spike level). Spike Level: nominal concentration added (mg L^−1^). SOB recovery: concentration recuperated in the MCP supernatant. %Rec.: percentage recovery, calculated as 100 × (SOB recovery/Spike Level). The mean concentration and percentage recovery were calculated from the HPLC analysis of the MCP supernatant (SOB). The recovery studies used five spike levels across three different matrices. The associated regression analysis used 15 data points (five spike levels × three matrix means).

**Table 6:** Determination of DMAC_SOB, MCP, and SFI in seed, skin, and wine matrices by cultivar and maceration technique.

Matrix	Cultivar	Technique	DMAC (orig., mg L^−1^)	MCP (mg L^−1^)	SFI (%)
seed	Marselan		52.73 ± 1.97 ^aA^	889 ± 14 ^aB^	5.9 ± 0.2 ^aA^
seed	Tannat		48.96 ± 2.49 ^aB^	883 ± 4 ^bB^	5.6 ± 0.3 ^aA^
skin	Marselan		20.01 ± 3.66 ^aB^	1664 ± 227 ^aA^	1.2 ± 0.3 ^aB^
skin	Tannat		28.84 ± 5.70 ^aB^	1666 ± 232 ^aA^	1.7 ± 0.1 ^aB^
wine	Marselan	Enzymes	89.77 ± 4.76 ^bA^	1438 ± 65 ^aB^	6.3 ± 0.1 ^bA^
wine	Marselan	Extended	108.10 ± 3.17 ^aA^	1445 ± 82 ^aA^	7.5 ± 0.4 ^aA^
wine	Tannat	Enzymes	70.07 ± 8.89 ^aB^	1728 ± 38 ^aA^	4.0 ± 0.5 ^aB^
wine	Tannat	Extended	65.20 ± 5.64 ^aB^	1395 ± 226 ^bA^	4.6 ± 0.0 ^aB^

Note: *n* = 3 for each matrix × cultivar (and technique, for wines). SFI (%) = (DMAC_SOB/MCP) × 100, with both DMAC_SOB and MCP expressed as mg·L−1 of (−)-epicatechin equivalents. Lowercase letters indicate significant differences between cultivars within a given matrix (seed or skin) or between winemaking techniques within a given cultivar (wines). Uppercase letters indicate significant differences between matrices within a cultivar, or between cultivars within the same winemaking technique (wines), according to the test of Tukey (*p* < 0.05). orig. denotes the concentration in the original sample (extract or wine), calculated after applying the dilution factor.

## Data Availability

The data supporting the findings of this study are available from the corresponding author upon reasonable request. Access is restricted because further, not-yet-published findings could be derived from these data.

## Supplementary Materials

The following supporting information can be downloaded at https://www.mdpi.com/article/10.3390/foods14244257/s1. Figure S1: Calibration Curve for the MCP assay. Calibration curve prepared using epicatechin standards across the 10–250 mg L^−1^ concentration range. Points are means (*n* = 3); Figure S2: Calibration Curve for direct DMACA measurements. Direct DMAC response for wines and grape extracts across 0–25 mg L^−1^ epicatechin equivalents. Points are means (*n* = 3); Figure S3: Epicatechin Calibration Curve for the High-Performance Liquid Chromatography (HPLC) determinations. Calibration curve established using High-Performance Liquid Chromatography (HPLC). The figure shows the chromatographic response across the 0–100 mg L −1 range of (-)-Epicatechin equivalents. Points are means (*n* = 3); Table S1: Evaluation of precision and accuracy of the MCP calibration curve (back-calculated concentration data); Table S2: Evaluation of precision and accuracy of the direct DMAC calibration curve (back-calculated concentration data); Table S3: Evaluation of precision and accuracy of the HPLC calibration curve (back-calculated concentration data); Table S4: Inter-day DMAC_SOB calibration (0–6 mg L^−1^); Table S5: Intermediate precision and day to day agreement. Recovery by concentration level in supernatant after methylcellulose by matrix; Table S6: Regression analysis of spike concentration vs. recovered LMM flavan-3-ols quantified by DMAC reagent in MCP supernatant; Table S7: Regression Analysis of Spike Concentration vs. Recovered LMM Flavan-3-ols quantified by HPLC.

## Abbreviations

The following abbreviations are used in this manuscript:

MC	Methylcellulose (reagent/solution)
MCP	methylcellulose precipitation assay (procedure)
DMAC	Dimethylaminocinnamaldehyde
HPLC	High-Performance Liquid Chromatography
SOB	Supernatant
HMM	High-Molecular-Mass
LMM	Low-Molecular-Mass
SFI	Small Flavanol Index
R&D	Results and Discussion
SI	Supporting Information
