# Supplementary material for: Development and Validation of a Selective Method to Quantify Low-Molecular-Mass Flavan-3-ols in Grapes and Wines
Source: Foods. 2025 Dec 10;14(24):4257. doi: 10.3390/foods14244257 (PMC12731937; doi:10.3390/foods14244257)
Supplement: Supplementary file 1 [file foods-14-04257-s001.zip › foods-4003350-supplementary.pdf]

SUPPLEMENTARY MATERIAL

Article

Development and Validation of A Selective Method to Quantify Low-Molecular-Mass Flavan-3-ols in Grapes and Wines

Guzmán Favre [1,*], Gustavo González-Neves [1], Diego Piccardo [1], Yamila Celio-Ackermann [1], Florencia Pereyra-Farina [1], Alejandro Cammarota [1]

[1] Facultad de Agronomía, Universidad de la República, Avda. Garzón 780. C.P., 12900 Montevideo, Uruguay; comunicacion@fagro.edu.uy

* Correspondence: gfavre@fagro.edu.uy; Tel.: (+59899440438)

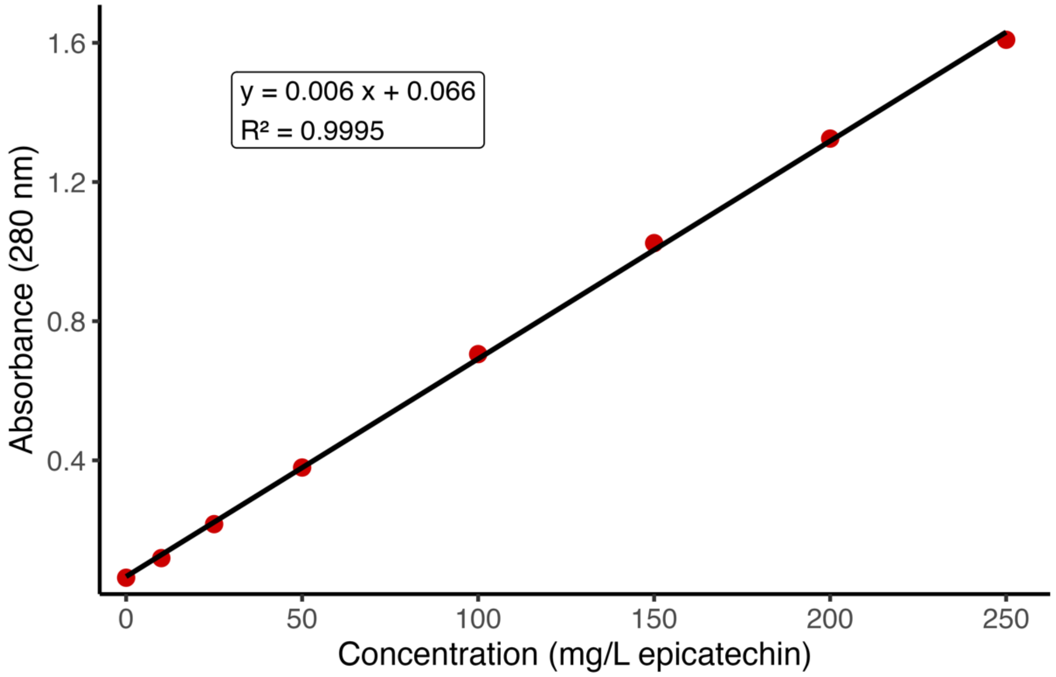

Figure S1. Calibration Curve for the MCP assay. Calibration curve prepared using epicatechin standards across the 10–250 mg L^{-1} concentration range. Points are means (n = 3).

Table S1. Evaluation of precision and accuracy of the MCP calibration curve (back-calculated concentration data).

Standard Concentrations	Mean calc. (mg L^{-1})	SD	RSD	Bias (%)
0	-0,53	0.025	40.57	NA
10	8,47	0.001	0.84	-15,29
25	24,08	0.001	0.26	-3,69
50	50,07	0.001	0.40	0,14
100	102,16	0.005	0.71	2,16
150	153,13	0.005	0.46	2,09
200	201,12	0.005	0.35	0,56
250	246,50	0.011	0.68	-1,40

Notes: For each concentration level 3 replicates were analyzed (n = 3); SD = Standard Deviation; RSD (%) = Relative Standard Deviation; Bias (%) = Relative Error, NA = Not Applicable (used where calculation involves division by zero). Bias (%) was calculated from back calculated concentrations, excluding the 0 mg L^{-1} level. Mean calc. (mg L^{-1}) = Mean concentration back-calculated using the calibration curve.

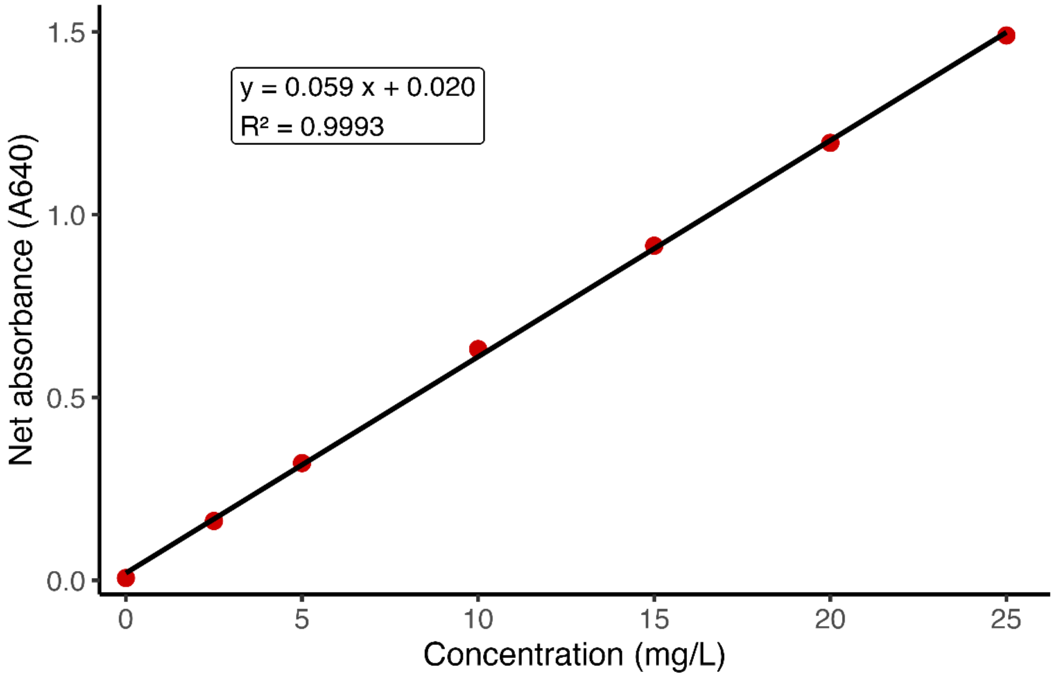

Figure S2. Calibration Curve for direct DMACA measurements. Calibration curve using (-)-Epicatechin standards ranging from 0–25 mg L^{-1} epicatechin equivalents. Points are means (*n* = 3).

Table S2. Evaluation of precision and accuracy of the direct DMAC calibration curve (back-calculated concentration data).

Standard Concentrations	Mean calc. (mg L⁻¹)	SD	RSD (%)	Bias (%)
0.0	-0,23	0.000	9.12	NA
2.5	2,41	0.003	1.88	-3,69
5.0	5,09	0.008	2.60	1,70
10.0	10,35	0.005	0.81	3,55
15.0	15,13	0.007	0.76	0,90
20.0	19,89	0.016	1.33	-0,54
25.0	24,86	0.017	1.16	-0,57

Notes: For each concentration level 3 replicates were analyzed (n = 3); SD = Standard Deviation; RSD (%) = Relative Standard Deviation; Bias (%) = Relative Error; NA = Not Applicable (used where calculation involves division by zero). Bias (%) was calculated from back calculated concentrations, excluding the 0 mg L^{-1} level. Mean calc. (mg L^{-1}) = Mean concentration back-calculated using the calibration curve.

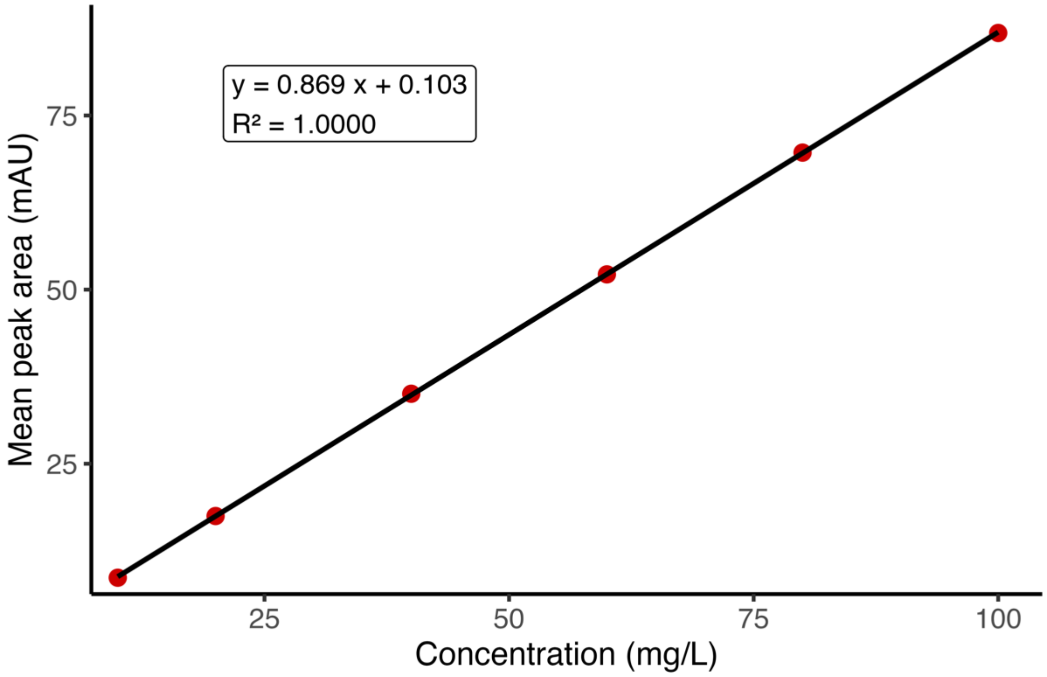

Figure S3. Epicatechin Calibration Curve for the High-Performance Liquid Chromatography (HPLC) determinations. Calibration curve established using High-Performance Liquid Chromatography (HPLC). The figure shows the chromatographic response across the 0–100 mg L^{-1} range of (-)-Epicatechin equivalents. Points are means (n = 3).

Table S3. Evaluation of precision and accuracy of the HPLC calibration curve (back-calculated concentration data).

Standard Concentrations	Mean calc. (mg L^{-1})	SD	RSD (%)	Bias (%)
10	9,82	0.039	0.45	-1,77
20	20,02	0.027	0.16	0,09
40	40,23	0.054	0.15	0,58
60	59,97	0.058	0.11	-0,05
80	80,09	0.099	0.14	0,11
100	99,87	0.060	0.07	-0,13

Notes: For each concentration level 3 replicates were analyzed (n = 3); SD = Standard Deviation; RSD (%) = Relative Standard Deviation. Bias (%) = Relative Error. Bias was calculated from back-calculated concentrations. Mean calc. (mg L^{-1}) = Mean concentration back-calculated using the calibration curve.

Table S4. Inter-day DMAC_SOB calibration (0–6 mg L^{-1})

Parameter	Mean ± SD (SOB)
**Slope (b1)**	0.062 ± 0.001 A.U. (mg·L^{-1})$^{-1}$
**Intercept (b0)**	0.009 ± 0.005 A.U.
**Residual SD**	0.01012 ± 0.00574 A.U.
**R^2**	0.99466 ± 0.00653
**LOD**	0.53978 ± 0.29940 mg·L^{-1}
**LOQ**	1.63569 ± 0.90727 mg·L^{-1}

Notes: Calibration curves were built from three replicate standard sets (0, 1, 2, 3, 4.5, 6 mg L^{-1}) using net absorbance at 640 nm. Parameters reported as mean ± SD across replicates: slope (A.U.·(mg L^{-1})$^{-1}$), intercept (A.U.), residual SD (A.U.; σ of residuals), and R^2 (coefficient of determination). LOD = 3.3·σ/slope and LOQ = 10·σ/slope (mg L^{-1}).

Table S5: Intermediate precision and day to day agreement. Recovery of the DMAC assay applied to MCP supernatants by matrix.

Matrix	Spike Added (mg L^{-1})	Recuperated (mg L^{-1}) (Mean ± SD)	Recovery (%) (Mean ± SD)	n
**skin**	7.5	6.1 ± 1.6	81.4 ± 21.0	4
**skin**	30	31.0 ± 1.1	103.2 ± 3.7	4
**seed**	7.5	7.4 ± 1.9	98.9 ± 25.0	3
**seed**	30	30.9 ± 5.1	103.1 ± 16.9	4
**wine**	7.5	6.8 ± 1.1	90.2 ± 14.8	4
**wine**	30	34.5 ± 2.2	114.9 ± 7.4	4

Notes: Added, nominal spike level (mg L^{-1}). Recuperated (mg L^{-1}) is the concentration recovered. Recovery %, mean and standard deviation of percent recovery, computed as 100 × C Recuperated/Added (%). An outlier was removed from seed, 7.5 spike level (n = 3). MCP, methylcellulose precipitation assay; DMAC, dimethylaminocinnamaldehyde

Table S6. Regression analysis of spike concentration vs. recovered LMM flavan-3-ols quantified by DMAC reagent in MCP supernatant.

Matrix	slope	intercept	R^2
**Skin**	1.002	-0.56	0.9752
**Seed**	1,011	-2.074	0.9735
**Wine**	0.94	5.55	0.9836

Notes: Slope (b), regression slope of recovered vs. added concentration; Intercept (a), regression intercept (mg L^{-1}); R^2, coefficient of determination; n = 5 (spike levels per matrix). MCP, methylcellulose precipitation assay; LMM, low molecular mass flavanols; DMAC, dimethylaminocinnamaldehyde.

Table S7. Regression Analysis of Spike Concentration vs. Recovered LMM Flavan-3-ols quantified by HPLC.

Matriz	Slope	Intercept	R^2	n
**seed_sob**	1.0061	-0.027	0.9996	6
**skin_sob**	1.0022	0.2372	0.9997	6
**wine_sob**	1.5583	-1.1118	0.9997	6

Notes: Slope (b). regression slope of recovered vs. added concentration; Intercept (a). regression intercept (mg L^{-1}); R^2. coefficient of determination. sob, MCP supernatant. LMM, low molecular mass flavanols.
